# KSHV Genome in Saliva, Whole Blood and Kaposi's Sarcoma Biopsy Specimens in Republic of Congo: Phylogenetic Analysis and an APOBEC3B Mutational Signature

**DOI:** 10.1002/jmv.70933

**Published:** 2026-04-30

**Authors:** Gervillien Arnold Malonga, Valentin Leducq, Antoine Fauchois, Dimitry Moudiongui Mboungou Malanda, Patrina Joseph Iloukou Mayakia, Juthèce Private Malanda‐Kiminou, Ferdinand Emaniel Brel Got, Fabien Gaël Mouamba, Vincent Calvez, Jean Felix Peko, Anne‑Geneviève Marcelin, Aude Jary

**Affiliations:** ^1^ Sorbonne Université, INSERM, Institut Pierre Louis d'Epidémiologie et de Santé Publique, Assistance Publique – Hôpitaux de Paris (AP‐HP), Hôpitaux Universitaires Pitié‐Salpêtrière ‐ Charles Foix, Laboratoire de Virologie Paris France; ^2^ Faculté des Sciences de la Santé Université Marien Ngouabi Brazzaville Republic of Congo; ^3^ Service d'Anatomie et Cytopathologie Centre Hospitalier Universitaire de Brazzaville Brazzaville Republic of Congo

**Keywords:** APOBEC3B mutations, Congo, KSHV, phylogenetic analysis, whole‐genome sequencing

## Abstract

Kaposi's sarcoma‐associated herpesvirus (KSHV) has been associated with a variety of diseases and is endemic in Sub‐Saharan Africa. We performed KSHV‐whole‐genome analysis and search for APOBEC‐editing to describe genetic diversity in samples issued from an endemic region. We included 15 Congolese samples including 12 saliva and whole blood samples of people living with HIV and 3 KS biopsies samples, to perform KSHV qPCR and NGS Illumina followed by KSHV typing on ORFs K1 and K15. Furthermore, we looked for APOBEC3B‐mutations in KSHV‐whole‐genome sequences. KSHV viral load was detectable in all samples and ranged from 374 to 72799 copies/10^6^ cells. NGS allowed us to have 8 full sequences out of the 15 samples analyzed. Phylogenetic analysis on KSHV‐whole‐genome resulted in distinct phylogenetic clustering between the genomic sequences of Congolese KSHV and those derived from Western countries. KSHV ORF‐K1 subtypes A5, B and C were identified. On KSHV ORF‐K15, only the P subtype was observed. We found virtually no APOBEC3B‐induced mutations in KSHV genome. KSHV sequences from Congo samples present phylogenetic particularities but remain close to the whole genome sequences found in Sub‐Saharan Africa. We were able to observe, using bioinformatics tools, only very few mutations due to APOBEC3B on KSHV genome.

**Abreviations**
APOBEC: apolipoprotein B messenger RNA editing enzyme, catalytic polypeptide‐like
KSHV: Kaposi's sarcoma‐associated herpesvirus
ORF: Open Reading Frame
SSA: Sub‐Saharan Africa

## 1 Introduction

Kaposi Sarcoma‐associated Herpesvirus (KSHV) is the etiological agent of Kaposi's sarcoma (KS), Primary Effusion Lymphoma (PEL) and some cases of Multicentric Castleman's Disease (MCD) [1]. However, the majority of individuals infected with KSHV do not develop associated diseases [2]. Four epidemiological forms of Kaposi's sarcoma are recognized [3]: (i) The AIDS‐associated Kaposi sarcoma, which affects individuals with human immunodeficiency virus (HIV) infection, (ii) the iatrogenic form, which affects patients undergoing immunosuppressive therapy, (iii) the African endemic form, which is most common in parts of Central, Southern and Eastern Africa, and (iv) the classic KS, which most often affects elderly males of Mediterranean or Eastern European areas [4]. Although KS is more common in immunocompromised patients living with HIV (PLWH) [5, 6] and may occur concurrently or sequentially with other KSHV diseases [7], the precise etiology of this complex pathology remains poorly understood. Also, Sub‐Saharan Africa (SSA) remains the most relevant geographic region for KSHV infection [8].

KSHV is a human gammaherpesvirus double‐stranded DNA genome of approximately 170 kb showing a high conservation with up to 99% sequence identity between viral strains [9]. The 5' end of the KSHV genome encodes the hypervariable K1 gene, with up to 30% amino acid variability, resulting in seven major K1 subtypes, A to F [10], whose distribution is associated with ethnicity and geography [11]. The 3' terminus of viral genome encodes the K15 gene, which supports a further categorization of KSHV strains into P (predominant), M (minor) or N alleles, with up to 70% sequence identity divergence at the amino acid level [12]. Some phylogenetic studies have used the highly conserved central region of the KSHV genome with 9 discrete loci and lower levels of variation than K1 and K15, to characterize subtypes, while others have proposed a new classification of KSHV that takes into account the whole genome sequence of the virus [9, 10]. Next‐generation sequencing (NGS) methods have drastically increased the number of complete KSHV genomic sequences currently available worldwide. Unfortunately, in SSA where the burden of KS is endemic, only four countries namely Zambia, Uganda, Cameroon and Malawi have reported complete or nearly complete genome sequences of KSHV [9, 10, 11, 13].

During infection, several events can occur as a result of the host immune response, including genetic recombination and APOBEC mutations [14, 15, 16]. The apolipoprotein B messenger RNA editing enzyme, catalytic polypeptide‐like (APOBEC) family of DNA cytosine deaminases provides a broad and overlapping defense against viral infection. These proteins bind RNA and single‐stranded DNA and produce C to T base modifications through their cytidine deaminase activity. Until now, the mutation rate of herpesvirus has been estimated to be low, with only one error per 1‐ to 100‐ million nucleotides replicated [17]. However recent works provide evidence that, even with their double DNA‐stranded, several human herpesvirus genomes are potential substrate for cellular APOBEC enzymes and may contribute to the variability of their genomes overtime, as well as cancer development [18, 19, 20].

We conducted this study with the hypothesis that whole genome subtypes of KSHV described in other SSA countries would also circulate in the Congolese population. We studied the molecular diversity in the left (ORF‐K1) and right (ORF‐K15) extremities as well as the whole KSHV genome. We also looked for APOBEC3B mutations to determine which KSHV genes might be affected by the APOBEC‐editing activity.

## 2 Materials and Methods

### 2.1 Sample Collection

Saliva and whole blood samples were collected from PLWHV at PLWH surveillance centers, whom were asymptomatic for any KSHV diseases, as previously reported [8]. KS biopsy specimens were retrieved from the pathological anatomy and cytology department in Brazzaville University Hospital Center.

### 2.2 | Ethics Approval and Consent to Participate

This study was reviewed and approved by the Health Sciences Research Ethics Committee (CERSSA) of the Ministry of Scientific Research and Technological Innovation (Approval No. 222/MRSIT/IRSSA/CERSSA), which is the Institutional Review Board (IRB) for the approval of studies involving human subjects in the Republic of Congo. All clinical samples were de‐identified and analyzed anonymously. All patients provided written informed consent and procedures were carried out in accordance with ethical principles of the Declaration of Helsinki.

### 2.2 Samples DNA Extraction and KSHV Quantification

Saliva and whole blood DNA were extracted using the NucliSens® easyMAG® kit (BIOMERIEUX SA, FR) according to manufacturer's instructions. From formalin‐fixed paraffin‐embedded (FFPE) biopsies, total DNA was extracted with the QIAamp DNA FFPE Tissue kit according to the manufacturer's protocol (Qiagen). The extracted DNA was used for quantification of KSHV in saliva, whole blood and KS biopsy, as previously described [21].

### 2.3 KSHV Whole‐Genome Sequencing, Assembly and Phylogenic Analysis

Whole genomes from 15 samples with detectable KSHV‐DNA were sequenced using the NextSeq. 500 Illumina® 150 system after hybrid‐capture for viral genome enrichment, as previously described [22]. As part of the bioinformatic analysis, we first performed quality control on the Illumina output data, and trimmed reads. Then, we aligned the reads against KSHV GK18 reference genome (NC_009333) using Bowtie2 program. We called variants and obtained whole genome (WG‐KSHV) consensus sequences using Bcftools [23]. When depth sequencing was insufficient ( < 10X), unresolved nucleotides were identified as N in the sequence (Supporting method). Multiple alignments of WG‐KSHV sequences and K1 and K15 nucleotide extracted sequences from WG‐KSHV consensus sequences were performed using MAFFT with reference sequences issued from the NCBI database. These reference sequences included all the subtypes and variants currently known, i.e. A, B, C, D, E and F, which have been identified in different regions of the world. Phylogenetic trees for K1, K15 and WG‐KSHV were built with a likelihood approach, using the IQ‐TREE program and 1000 bootstraps replicates.

### 2.4 Identification of APOBEC G‐to‐A and C‐to‐T Mutation Sequences

For APOBEC mutations analysis, we performed a multiple sequence alignment of each consensus with the KSHV GK18 reference genome (NC_009333). Then, the APOBEC3B induced mutations (C > T or G > A) were determined with an in‐house pipeline. To directly analyze the context of putative APOBEC3B‐mediated changes, we exclusively analyzed the context of T**C** or of **G**A dinucleotides.

### 2.5 Statistical Analysis

Categorical variables were expressed as number and percentage and continuous variable as median and interquartile [IQR]. Non‐parametric tests, i.e., Fisher *t* test, Kruskal‐Wallis test and Mann‐Whitney U test, were used to compare categorical and continuous variables, respectively, and the Spearman rank test to evaluate the correlation between APOBEC3B frequency and KSHV‐DNA viral load. The significance was established with a *p*‐value < 0.05.

## 3 Results

### 3.1 Characteristics of the Patients

In this study, we analyzed the diversity of WG‐KSHV, ORF‐K1 and ORF‐K15 genes acquired from 15 Congolese samples including 3 KS FFPE biopsies, 3 whole blood samples and 9 saliva samples. Six patients were males (40%), with a median [IQR] age of 42 [29–54] years. According to HIV status, 14 (93%) were positive. The antiretrovials (ARVs) regimen most used was TDF‐FTC‐EFV (Tenofovir‐Emtricitabine‐Efavirenz). Clinically, most of the patients were asymptomatic while three patients had KS disease, of whom 2 had an AIDS‐associated form of KS (Table 1). KSHV viral load was detectable in all our samples (ranging from 374 to 72,799 copies/10^6^ cells) (Table 2).

**Table 1:** Demographic and clinical information's of the 15 patients included in the study.

Sample ID	Gender	Age (y)	HIV status	ART	Clinical presentation
1	F	46	Positive	TDF‐FTC‐EFV	AIDS‐associated KS
2	M	46	Positive	TDF‐FTC‐EFV	asymptomatic
3	F	54	Positive	AZT‐3TC‐NVP	asymptomatic
4	F	30	Positive	AZT‐3TC‐NVP	asymptomatic
5	M	26	Positive	TDF‐FTC‐EFV	asymptomatic
6	F	42	Positive	TDF‐FTC‐EFV	asymptomatic
7	F	29	Positive	LPV/r‐3TC‐ABC	asymptomatic
8	F	65	Positive	TDF‐FTC‐EFV	asymptomatic
9	F	29	Positive	LPV/r‐3TC‐ABC	asymptomatic
10	M	30	Positive	AZT‐3TC‐NVP	asymptomatic
11	F	30	Positive	TDF‐FTC‐EFV	asymptomatic
12	M	45	Positive	TDF‐FTC‐EFV	asymptomatic
13	M	61	Positive	AZT‐3TC‐NVP	AIDS‐associated KS
14	M	23	NA^a^	NA	KS ND^b^
15	F	54	Positive	TDF‐FTC‐EFV	asymptomatic

*Note:* ^a^Not available; ^b^KS form Not Determined.

Abbreviations: 3TC, Lamivudine, Epivir; ABC, Abacavir; AZT, Zidovudine, Retrovir; D4T, Stavudine; EFV, Efavirenz; FTC, Emtricitabine; LPV/r, Lopinavir/ritonavir; NVP, Nevirapine; TDF, Tenofovir disoproxil fumarate.

**Table 2:** Summary of KSHV viral load and typing from the 15 patients included in the study.

Sample ID	KSHV genome size (bp)	Coverage (%)	Sample source	Sample year	KSHV Viral load (copies/10^6^ cells)	KSHV‐typing (classification)^b^	K1/K15 subtype
1	60,096	44	KS biopsy	2012	72,799	—	‐/‐
2	124,307	90	Whole blood	2019	374	—	‐/‐
3	133,529	97	Whole blood	2019	429	P3	B1/P
4	135,977	99	Saliva	2019	436	P3	B1/P
5	134,721	98	Saliva	2019	894	P3	B1/P
6	121,664	88	Saliva	2019	901	—	‐/‐
7	125,517	91	Saliva	2019	905	—	B1/‐
8	121,195	88	Saliva	2019	1,817	—	‐/‐
9	137,069	99	Whole blood	2019	2,350	—	B1/P
10	137,260	99	Saliva	2019	3,727	P3	A5/P
11	129,720	94	Saliva	2019	4,839	—	‐/‐
12	81,313	59	Saliva	2019	5,063	—	B1/‐
13	138,037	100	KS^a^ biopsy	2021	7,879	P3	B1/P
14	138,014	99	KS biopsy	2021	51,392	P3	B1/P
15	135,374	98	Saliva	2019	67,636	M1	C7/P

^a^ Kaposi sarcoma.

^b^ Proposal based on whole genome data [9].

### 3.2 Sequencing Performance

In total after reads trimming, the median number of reads mapped to KSHV genome was 13,220 [2,170–63,947], with a median coverage of 97 [44–99] and a median sequencing depth of 11 [2–61]. Of the 15 samples sequenced, 9 (53%) achieved a good coverage (> 90%) with a good mean sequencing depth, i.e. > 10‐fold at the whole genome level and had uniform read coverage (see Supporting Figure 1). These nine samples could be used for further analysis. One additional sample was used for K1 phylogenetic analysis only, as the reads mapped strictly to the ORF‐K1 region (sample 7). The samples for which sequencing failed were mainly oral swabs (4/6) and the median of KSHV viral load was 1,359 [300–4,895] copies/10^6^ cells *versus* 2350 [665–29,636] copies/10^6^ cells for those with effective whole genome sequencing (*p* = *0.38*). Interestingly, the KSHV genomes from FFPE tissues, i.e. samples 13 and 14, had a larger size and read coverage than those from the other specimens (saliva and whole blood) (Table 2). However, the third FFPE sample (sample 1) could not be used due to poor coverage and depth sequencing, although KSHV viral load was quite high (72,799 copies/10^6^ cells).

### 3.3 ORF‐K1 and ORF‐K15 Phylogenetic Analysis

**Figure 1:**
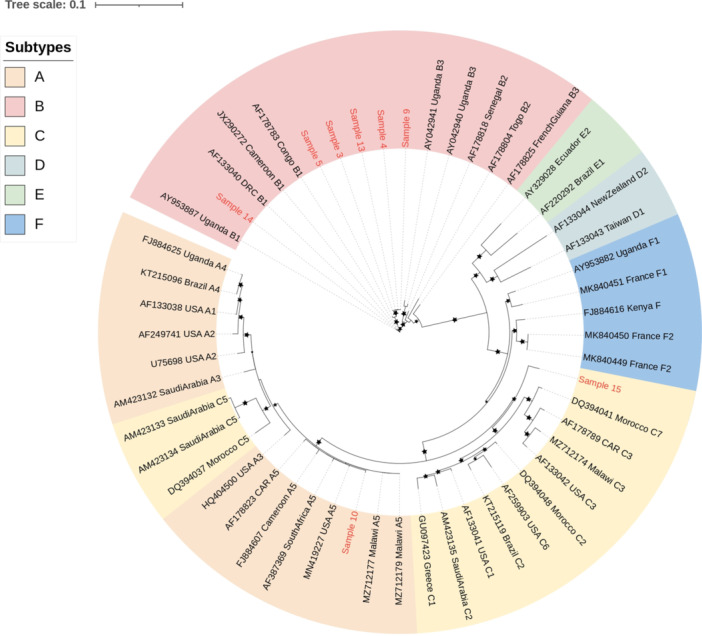
Maximum likelihood phylogenetic tree constructed with ORF‐K1 nucleotide sequences issued from saliva and whole blood of patients included in the study as well as 45 sequences issued from the NCBI database. *The patients' sequences are shown in red, and the reference sequences are shown in black. Stars represent nodes with bootstrap support* > *70% (bootstrap analysis with 1000 replicates). GenBank reference sequence accession numbers and their countries are shown in the figure. GenBank new reference sequence accession numbers were as follows: Sample 3: PV051112; Sample 4: PV051113; Sample 7: PV051114; Sample 9: PV051115; Sample 12: PV051116; Sample 13: PV051117; Sample 14: PV051118*.

ORF‐K1 amplification was successful for 8 sequences with full coverage for this gene and 2 additional samples with partial sequences, i.e. samples 7 and 12 (excluded from the phylogenic tree), allowing subtyping (*n* = 10). Subtype B was the most prevalent [8/10 (80%)], followed by subtype A [1/10 (10%)] and subtype C [1/10 (10%)]. All B subtypes were B1 variants and were detected in saliva (50%), in whole blood (25%) and KS biopsies samples (25%), respectively. The A5 variant and subtype C were only found in saliva samples (Figure 1).

**Figure 2:**
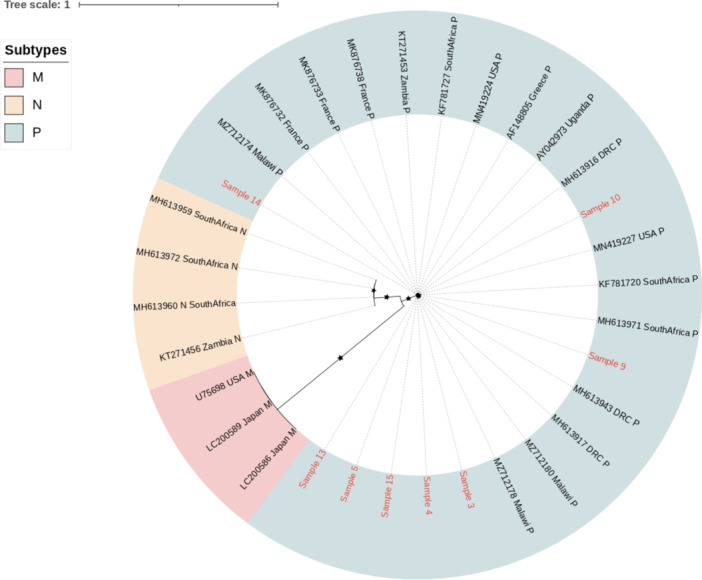
Maximum likelihood phylogenetic tree constructed with ORF‐K15 nucleotide sequences issued from saliva and whole blood of patients included in the study as well as 24 sequences issued from the NCBI database. *The patients' sequences are shown in red, and the reference sequences are shown in black. Stars represent nodes with bootstrap support* > *70% (bootstrap analysis with 1000 replicates). GenBank reference sequence accession numbers and their countries are shown in the figure. GenBank new reference sequence accession numbers were as follows: Sample 4: PV764557; Sample 5: PV764558; Sample 9: PV764559; Sample 10: PV764560; Sample11: PV764561; Sample 13: PV764562; Sample 14: PV764563; Sample 15: PV764564*.

Of the 14 successfully amplified samples, 8 sequences had a complete coverage of ORF‐K15. All samples tested belonged to ORF‐K15 subtype P, which was found in the following proportions: in saliva [4/8 (50%)], in whole blood [2/8 (25%)] and biopsies of MK [2/8 (25%)] (Figure 2). Reads were mapped against the KSHV reference genome (NC_009333). It is important to note that since this reference contains a subtype P K15 allele, our pipeline is specifically tailored for this subtype, and other K15 alleles (e.g., subtype M) were not assessed.

### 3.4 WG‐KSHV Variability

**Figure 3:**
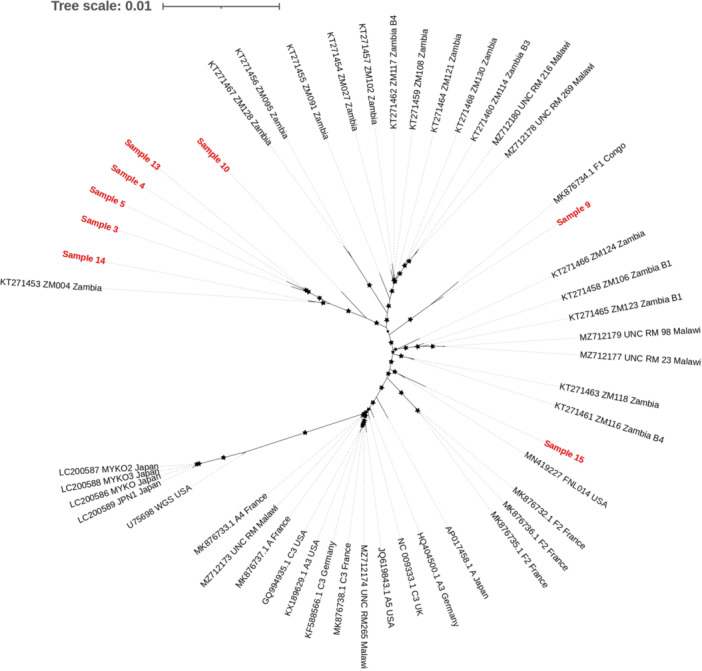
Unrooted maximum likelihood phylogenetic tree constructed with KSHV whole nucleotide sequences issued from saliva and whole blood of 8 patients included in the study as well as 42 sequences issued from the NCBI database. *The patients' sequences are shown in red, and the reference sequences are shown in black. Stars represent nodes with bootstrap support* > *70% (bootstrap analysis with 1000 replicates). GenBank reference sequence accession numbers and their countries are shown in the figure. GenBank reference sequence accession numbers were as follows: Sample 3: PV764565; Sample 4: PV764566; Sample 5: PV764567; Sample 9: PV764568; Sample 10: PV764569; Sample 13: PV764570; Sample 14: PV764571; Sample 15: PV764572*.

To determine the degree of variability in the WG‐KSHV genomes, we performed a phylogenetic analysis using reference sequences published from different parts of the world (Figure 3). Previously published Western and Japanese samples of WG‐KSHV (i.e., Europe, Japan, USA) clustered together; separately from the African samples (i.e. Zambia, Malawi and Congo). Isolates from Zambia, Malawi and Congo appear to form two distinct subgroups with the same origin. Most of our samples (6/8) clustered together and were close to the sequences from the African region. On the other hand, the 2 remaining samples were phylogenetically close to the MN419227 FNL014 and MK876734.1 sequences from the USA and from a women living in France but originated from Congo, respectively (Figure 3). Specifically, the sample 9 clustered with the F1 (MK876734.1) sequence which is the African variant of the subtype F (variant F1) and the sample 15 clustered with the A5 sequence (MN419227 FNL014) which is an African variant that is very common in sub‐Saharan Africa. In addition, we observed no distinct clustering patterns between genomes isolated from saliva compared to genomes isolated from whole blood and KS biopsies or between samples from asymptomatic and KS patients.

### 3.5 APOBEC Mutations

APOBEC3B editing was analyzed in WG‐KSHV nucleotide sequences with a coverage higher than 90% at a depth sequencing above 10X (*n* = 9). In total, 2907 C > T and G > A were found, of whom 670 were included in TC or GA motifs (Table 3). A median of 171 [66–190] APOBEC3B editing per nucleotide sequence was found, of whom 40 [18–42] included in TC or GA motifs. The percentage of genome affected by APOBEC3B editing was limited (0.029% [0.014%–0.031%]) and was distributed throughout the genome (Supporting Table 2). The frequency of APOBEC3B editing was different between saliva, whole blood and KS biopsies (*p* = 0.02) with a lower rate in saliva compared to whole blood and KS biopsies (0.024% *vs.* 0.030% and 0.032% in median, respectively). The frequency of APOBEC3B editing was not correlated with KSHV DNA viral load (r = 0.12, *p* = 0.62). Nevertheless, APOBEC3B‐compatible mutations were distributed throughout the viral genome and affects almost exclusively coding regions. Several ORFs showed a notable accumulation of editing events, notably ORF63/ORF64, ORF25–ORF27, ORF56/ORF57, and ORF48/ORF47. APOBEC editing were also observed in genes involved in latency and immune evasion, such as K1, K5, K7, K15, and vIRF genes, as well as LANA‐1 (ORF‐73), v‐FLIP (ORF71) and v‐Cyclin (ORF72) (Supporting Table 2).

**Table 3:** Summary of APOBEC3 mutational signature in WG‐KSHV nucleotide sequences with a coverage above 90% and depth sequencing above 10X.

Sample ID	APOBEC mutations	No	APOBEC mutations in specific motifs	No (%)
3	C – > T	189	TC – > TT	37 (0.028)
3	G – > A	178	GA – > AA	41 (0.031)
4	C – > T	169	TC – > TT	36 (0.027)
4	G – > A	150	GA – > AA	29 (0.021)
5	C – > T	212	TC – > TT	41 (0.030)
5	G – > A	190	GA – > AA	43 (0.032)
7	C – > T	95	TC – > TT	23 (0.018)
7	G – > A	66	GA – > AA	18 (0.014)
9	C – > T	192	TC – > TT	49 (0.036)
9	G – > A	167	GA – > AA	40 (0.029)
10	C – > T	166	TC – > TT	41 (0.030)
10	G – > A	122	GA – > AA	30 (0.022)
13	C – > T	201	TC – > TT	42 (0.031)
13	G – > A	176	GA – > AA	40 (0.029)
14	C – > T	204	TC – > TT	47 (0.034)
14	G – > A	172	GA – > AA	49 (0.036)
15	C – > T	139	TC – > TT	30 (0.022)
15	G – > A	119	GA – > AA	34 (0.025)

## 4 Discussion

In this study, we utilized NGS methodology to sequence WG‐KSHV and to examine the molecular diversity of its complete genome, as well as of the K1 and K15 genes, with the objective of characterizing potential subtypes from saliva, whole blood and KS biopsies collected in SSA. Subsequently, a search was conducted for mutations induced by APOBEC3B activity, operating under the hypothesis that human APOBEC3 proteins play a role in genome variability and evolution [24]. The WG‐KSHV data provide new evidence on the uniqueness of KSHV strains in African populations. Moreover, whole‐genome sequence analysis corroborated earlier findings concerning the conservation of the KSHV genome and the restriction of APOBEC activity to KSHV genome diversity.

The present study reports the sequencing and analysis of 15 unique Congolese KSHV genomes isolated from saliva, whole blood and KS tumors in PLWH. A breakthrough has been achieved through the successful detection of KSHV‐DNA and subsequent whole genome sequencing even at moderate rate of viral load in saliva and whole blood samples from asymptomatic patients. The median viral loads were 9,580 copies/10^6^ cells and 1,051 copies/10^6^ cells in the saliva and whole blood, respectively *versus* 44,023 copies/10^6^ cells in FFPE tissues of KS lesions. In accordance with the findings of the Ugandan KS study, it has been demonstrated that levels of KSHV viral load are significantly lower in saliva and whole blood compared to tumor biopsies or tumor‐derived cell lines. This observation may provide a rationale for the scarcity of WG‐KSHV from healthy individuals [9]. K1 subtypes A5 and B are very common in sub‐Saharan Africa [25, 26], among Bantu peoples (ethnic groups of Africa who have very similar languages and customs and inhabit a geographical area extending from central to southern Africa) [12, 26]. The high predominance of subtype B K1 has previously been reported in Congo [8]. While the type P/type M/type N classification, based on variation in the K15 gene have been identified as subtypes circulating on the African continent, we only identified the subtype P of the KSHV K15 allele in our samples. Our results are in contrast to those of *Lacoste* et al. who reported in their cohort of patients from Central Africa the exclusive circulation of KSHV K15 subtype M [25, 27].

A comparison was made between the KSHV sequences under investigation and those previously published from Western countries [9, 13, 28] as well as from a region where KS is endemic, particularly a part of southern Africa recognized as the Kaposi Belt [10, 29]. Multiple alignment of the eight newly sequenced WG‐KSHV demonstrated a good genome‐wide coverage and exhibited distinct phylogenetic clustering between the genomic sequences of the Zambian and Congolese KSHV isolates and those from Western countries. *Olp* et al. also reported a phylogenic distinction between African KSHV sequences and those observed in Western countries [10]. This finding provides a plausible explanation for the presence of intra‐variability within the entire KSHV genome, which is specific to the virus itself and also varies among populations and ethnic groups. In their report, *Sallah* et al. present a new nomenclature for KSHV, with the new system considering the entire genome. Despite the fact that it is not internationally recognized or accepted by the scientific community in the same way as the original K15 classification, this nomenclature system names the Western KSHV genomes as P1 (USA, Greece) and M1 (Japan) and the Sub‐Saharan KSHV genomes as P1 and P2 (Zambia and Uganda) [9]. Although the research by Sallah et al. was limited by the overrepresentation of mainly African genomes, we report in this study new but closed variants to other KSHV genomes from sub‐Saharan Africa. Indeed the KSHV genomes were found to cluster together in the phylogenetic tree (see Figure 3), thereby forming a distinct subgroup alongside sequences derived from Zambia and Malawi.

Only gammaherpesviruses, EBV and KSHV, interact with A3B, notably through the interactions of the large ribonucleotide reductase (RNR) viral subunits with A3B [20]. RNR encoded by KSHV ORF‐61 interacts directly with A3B, inhibits its DNA deaminase activity, and re localizes it from the nuclear to the cytoplasmic compartment [30]. The importance of this A3B neutralization mechanism is highlighted by KSHV ORF‐61 causing an accumulation of C/G‐to‐T/A mutation of the A3B signature. The fact that we found very few A3B‐induced mutations in the KSHV genome (no more than 5 mutations per gene) means that the KSHV genome is well conserved and that KSHV ORF‐61, through RNR, efficiently reprimands, relocates, and inhibits human A3B. This supports the observation that KSHV exhibits a high degree of sequence conservation with a remarkably low error rate, a feature that appears to be common to double‐stranded DNA herpesviruses [17, 20, 31, 32]. EBV also exhibits a preferential accumulation of APOBEC‐induced mutations in certain latency and replication genes [33]. Similarly, the distribution of mutations observed in KSHV affects several ORFs involved in latency and immune evasion, notably the vIRF and K genes, as well as the 3 mains drivers of the latency: LANA‐1, v‐Flip and v‐Cyclin. These observations suggest that the latency or reactivation phases, during which the KSHV viral genome is maintained in a nuclear episomal form, could constitute prime windows for APOBEC‐editing. Genetic recombination may play a more important role than APOBEC mutations in KSHV genome variability. It not only allows for the generation of new genetic combinations and promotes the emergence of viral subtypes, but it also plays a role in genomic rearrangements, thus influencing the evolution and potentially the pathogenicity of this oncogenic herpesvirus [34, 35].

However, this study has certain limitations: (i) Several of our samples have an KSHV viral load that is not high enough to obtain genomes of significant size using Illumina sequencing. The low concentration of viral DNA directly impacted the efficiency of Illumina sequencing. Below a certain viral load threshold, enrichment and capture of the entire genome become suboptimal, preventing the acquisition of genomes of significant size and limiting the exhaustive characterization of the genetic diversity of entire viral genomes compared to other studies. This technical constraint explains the fragmentation of certain assemblies and highlights the inherent difficulty of studying the KSHV genome directly from patient samples, especially from FFPE tissue. (ii) As shown in Supporting Figure 1, the coverage and sequencing depth of our study sequences are so disparate that the identification of APOBEC mutations may have been underestimated. This heterogeneity in sequencing depth may have led to an underestimation of APOBEC‐associated mutational signatures, particularly in genomic regions with low coverage which could not be analyzed. Given that reliable identification of APOBEC‐induced mutations requires sufficient and relatively uniform coverage, the observed disparities likely reduced our sensitivity to detecting rare or region‐specific mutational events, as insufficient coverage and depth sequencing in certain genomic regions reduces the statistical power needed to distinguish authentic APOBEC‐induced mutations from sequencing background noise. (iii) Very few patients were included in our study, which probably explains why the statistical results do not have rich significance. The small sample size inevitably reduced the statistical power of our analyses and could explain the lack of robust statistical significance observed for several associations. Although the trends identified are biologically consistent, they should be interpreted with caution, as they may not fully reflect the variability of KSHV genomic evolution and require validation in a larger multicenter cohort to confirm their epidemiological and clinical relevance. (iv) We have very little complete genome with suboptimal depth/coverage, hence the presence in our NGS consensus sequences of areas of unknown nucleotide (N) positioning as well as degenerate nucleotides. As a result, the consensus sequences generated by next‐generation sequencing (NGS) contained regions of unresolved nucleotides (marked “N”) as well as degenerate bases. These ambiguities may have affected subsequent analyses, including phylogenetic reconstruction and mutation frequency estimation, and further highlight the technical difficulties associated with sequencing low‐abundance viral genomes. (v) Our bioinformatic pipeline is the reliance on the NC_009333 reference genome for K15 mapping. Since NC_009333 carries a subtype P allele, our study specifically characterizes K15 subtype P variants. Given the significant evolutionary distance between K15 subtypes, other alleles (notably subtype M) could not be assessed using this methodology. Nevertheless, this choice does not invalidate our results because the K15 subtype M remains less dominant in sub‐Saharan African populations. Future studies employing a multi‐reference alignment strategy will be required to fully capture the K15 diversity in this population.

In conclusion, our results highlight the need for comprehensive genomic nomenclature encompassing the entire KSHV genome to characterize its diversity and variability. Although the K1 and K15 ends of KSHV exhibit higher levels of polymorphism than the rest of the genome, they alone are not sufficiently representative to measure the diversity of the KSHV genome. Furthermore, the use of bioinformatics tools has revealed very few mutations caused by APOBEC3B in the KSHV genome. Nevertheless, many key genes, including those involved in latency and immune evasion (vIRF genes and several K genes), are affected by these mutations. Although this study enriches the diversity of quasi‐complete KSHV genome sequences, our results highlight the need for future research. The use of larger cohorts and improved viral enrichment strategies will be crucial for accurately mapping the virus's genomic diversity. This will allow us to decipher APOBEC‐related mutational processes, the mechanisms of APOBEC3B enzyme neutralization by KSHV, and the impact of genetic recombination.

## Author Contributions

Gervillien Arnold Malonga, Vincent Calvez, Jean Felix Peko, Anne‑Geneviève Marcelin and Aude Jary designed and planned the study. Dimitry Moudiongui Mboungou Malanda, Patrina Joseph Iloukou Mayakia, Juthèce Private Malanda‐Kiminou, Ferdinand Emaniel Brel Got, Fabien Gaël Mouamba and Jean Felix Peko recruitment, provided participant care, acquired the data, preparation and mounting of slides for microscopic examination and histopathological diagnosis of Kaposi's sarcoma. Gervillien Arnold Malonga and Valentin Leducq conducted the experiments. Gervillien Arnold Malonga, Valentin Leducq, Antoine Fauchois and Aude Jary analyzed and interpreted the data. Jean Felix Peko, Anne‑Geneviève Marcelin and Aude Jary validated and supervised protocols. Gervillien Arnold Malonga and Aude Jary wrote the manuscript. Gervillien Arnold Malonga, Vincent Calvez, Jean Felix Peko, Anne‑Geneviève Marcelin and Aude Jary revised the manuscript. All the authors read and approved the final manuscript.

## Conflicts of Interest

The authors declare no conflicts of interest.

## Supporting information

Supporting Figure

Supporting Method

Supporting Table 1

Supporting Table 2

## Data Availability

The data that support the findings of this study are available from the corresponding author upon reasonable request. KSHV sequences information is available in GenBank referencing accession numbers PV051112‐PV051118 and PV764557‐PV764572.

## References

[1] Y. Kawaguchi , Y. Mori , and H. Kimura , ed., Human Herpesviruses. Springer Singapore, 2018. 1045, 10.1007/978-981-10-7230-7.

[2] E. M. Cornejo Castro , V. Marshall , J. Lack , et al., “Dual Infection and Recombination of Kaposi Sarcoma Herpesvirus Revealed by Whole‐Genome Sequence Analysis of Effusion Samples,” Virus Evolution 6, no. 2 (2020): veaa047. 34211736 10.1093/ve/veaa047 PMC7474928

[3] A. Jary , M. Veyri , A. Gothland , V. Leducq , V. Calvez , and A. G. Marcelin , “Kaposi's Sarcoma‐Associated Herpesvirus, the Etiological Agent of All Epidemiological Forms of Kaposi's Sarcoma,” Cancers 13, no. 24 (2021): 6208, 10.3390/cancers13246208. 34944828 PMC8699694

[4] P. E. Zeinaty , C. Lebbé , and J. Delyon , “Endemic Kaposi's Sarcoma,” Cancers 15, no. 3 (2023): 872, 10.3390/cancers15030872. 36765830 PMC9913747

[5] S. J. Coates and K. S. Leslie , “What's New in HIV Dermatology?,” F1000Research 8 (2019): 980, 10.12688/f1000research.16182.1. PMC6600856 31297183

[6] G. Arnold Malonga , D. M. Mboungou Malanda , and P. J. Iloukou Mayakia , “Epidemiological Data on the Histopathological Diagnosis of Kaposi's Sarcoma in Brazzaville, Republic of Congo,” International Journal of Health Sciences and Research 13, no. 5 (2023): 245–249, 10.52403/ijhsr.20230529.

[7] K. Lurain , M. N. Polizzotto , K. Aleman , et al., “Viral, Immunologic, and Clinical Features of Primary Effusion Lymphoma,” Blood 133, no. 16 (2019): 1753–1761, 10.1182/blood-2019-01-893339. 30782610 PMC6473499

[8] G. A. Malonga , A. Jary , V. Leducq , et al., “Seroprevalence and Molecular Diversity of Human Herpesvirus 8 Among People Living With HIV in Brazzaville, Congo,” Scientific Reports 11, no. 1 (2021): 17442, 10.1038/s41598-021-97070-4. 34465868 PMC8408137

[9] N. Sallah , A. L. Palser , S. J. Watson , et al., “Genome‐Wide Sequence Analysis of Kaposi Sarcoma‐Associated Herpesvirus Shows Diversification Driven by Recombination,” Journal of Infectious Diseases 218, no. 11 (2018): 1700–1710, 10.1093/infdis/jiy427. 30010810 PMC6195662

[10] L. N. Olp , A. Jeanniard , C. Marimo , J. T. West , and C. Wood , “Whole‐Genome Sequencing of Kaposi's Sarcoma‐Associated Herpesvirus From Zambian Kaposi's Sarcoma Biopsy Specimens Reveals Unique Viral Diversity,” Journal of Virology 89, no. 24 (2015): 12299–12308, 10.1128/JVI.01712-15. 26423952 PMC4665246

[11] V. A. Marshall , N. C. Fisher , C. A. Goodman , et al., “Systematic Analysis of Kaposi's Sarcoma (KS)‐Associated Herpesvirus Genomes From a KS Case‐Control Study in Cameroon: Evidence of Dual Infections but no Association Between Viral Sequence Variation and KS Risk,” International Journal of Cancer 151, no. 7 (2022): 1127–1141, 10.1002/ijc.34136. 35608873 PMC10043945

[12] G. S. Hayward and J. C. Zong Modern Evolutionary History of the Human KSHV Genome. In: Boshoff C., Weiss R. A., eds. Kaposi Sarcoma Herpesvirus: New Perspectives. Current Topics in Microbiology and Immunology. Springer; 2007:1‐42, 10.1007/978-3-540-34344-8_1. 17089792

[13] R. Moorad , A. Juarez , J. T. Landis , et al., “Whole‐Genome Sequencing of Kaposi Sarcoma‐Associated Herpesvirus (KSHV/HHV8) Reveals Evidence for Two African Lineages,” Virology 568 (2022): 101–114, 10.1016/j.virol.2022.01.005. 35152042 PMC8915436

[14] K. Butler and A. R. Banday , “APOBEC3‐mediated Mutagenesis in Cancer: Causes, Clinical Significance and Therapeutic Potential,” Journal of Hematology & Oncology 16, no. 1 (2023): 31, 10.1186/s13045-023-01425-5. 36978147 PMC10044795

[15] V. C. Vieira and M. A. Soares , “The Role of Cytidine Deaminases on Innate Immune Responses Against Human Viral Infections,” BioMed Research International 2013 (2013): 1–18, 10.1155/2013/683095. PMC3707226 23865062

[16] K. Cervantes‐Gracia , A. Gramalla‐Schmitz , J. Weischedel , and R. Chahwan , “APOBECs Orchestrate Genomic and Epigenomic Editing Across Health and Disease,” Trends in Genetics 37, no. 11 (2021): 1028–1043, 10.1016/j.tig.2021.07.003. 34353635

[17] A. Z. Cheng , S. N. Moraes , N. M. Shaban , et al., “APOBECs and Herpesviruses,” Viruses 13, no. 3 (2021): 390, 10.3390/v13030390. 33671095 PMC7998176

[18] K. Wakae , S. Kondo , H. T. Pham , et al., “EBV‐LMP1 Induces APOBEC3s and Mitochondrial DNA Hypermutation in Nasopharyngeal Cancer,” Cancer Medicine 9, no. 20 (2020): 7663–7671, 10.1002/cam4.3357. 32815637 PMC7571841

[19] I. Bobrovnitchaia , R. Valieris , R. D. Drummond , et al., “APOBEC‐Mediated DNA Alterations: A Possible New Mechanism of Carcinogenesis in EBV‐Positive Gastric Cancer,” International Journal of Cancer 146, no. 1 (2020): 181–191, 10.1002/ijc.32411. 31090066

[20] A. Z. Cheng , J. Yockteng‐Melgar , M. C. Jarvis , et al., “Epstein–Barr Virus BORF2 Inhibits Cellular APOBEC3B to Preserve Viral Genome Integrity,” Nature Microbiology 4, no. 1 (2019): 78–88, 10.1038/s41564-018-0284-6. PMC6294688 30420783

[21] F. Lallemand , N. Desire , W. Rozenbaum , J. C. Nicolas , and V. Marechal , “Quantitative Analysis of Human Herpesvirus 8 Viral Load Using a Real‐Time PCR Assay,” Journal of Clinical Microbiology 38, no. 4 (2000): 1404–1408, 10.1128/JCM.38.4.1404-1408.2000. 10747115 PMC86453

[22] A. Jary , V. Leducq , N. Desire , et al., “New Kaposi's Sarcoma‐Associated Herpesvirus Variant in Men who Have Sex With Men Associated With Severe Pathologies,” Journal of Infectious Diseases 222, no. 8 (2020): 1320–1328, 10.1093/infdis/jiaa180. 32282911

[23] A. BeltCappellino , V. Majerciak , A. Lobanov , J. Lack , M. Cam , and Z. M. Zheng , “CRISPR/Cas9‐Mediated Knockout and In Situ Inversion of the ORF57 Gene from All Copies of the Kaposi's Sarcoma‐Associated Herpesvirus Genome in BCBL‐1 Cells,” Journal of Virology 93, no. 21 (2019): e00628‐19, 10.1128/jvi.00628-19. 31413125 PMC6803266

[24] D. Forni , R. Cagliani , U. Pozzoli , and M. Sironi , “An APOBEC3 Mutational Signature in the Genomes of Human‐Infecting Orthopoxviruses,” mSphere 8, no. 2 (2023): e00062‐23, 10.1128/msphere.00062-23. 36920219 PMC10117092

[25] E. Etta , D. Alayande , L. Mavhandu‐Ramarumo , G. Gachara , and P. Bessong , “HHV‐8 Seroprevalence and Genotype Distribution in Africa, 1998–2017: A Systematic Review,” Viruses 10, no. 9 (2018): 458, 10.3390/v10090458. 30150604 PMC6164965

[26] T. Isaacs , A. B. Abera , R. Muloiwa , A. A. Katz , and G. Todd , “Genetic Diversity of HHV8 Subtypes in South Africa: A5 Subtype is Associated With Extensive Disease in AIDS‐KS,” Journal of Medical Virology 88, no. 2 (2016): 292–303, 10.1002/jmv.24328. 26174882

[27] V. Lacoste , J. G. Judde , J. Brière , et al., “Molecular Epidemiology of Human Herpesvirus 8 in Africa: Both B and A5 K1 Genotypes, as Well as the M and P Genotypes of K14.1/K15 Loci, Are Frequent and Widespread,” Virology 278, no. 1 (2000): 60–74, 10.1006/viro.2000.0629. 11112482

[28] R. Awazawa , D. Utsumi , H. Katano , et al., “High Prevalence of Distinct Human Herpesvirus 8 Contributes to the High Incidence of Non‐Acquired Immune Deficiency Syndrome‐Associated Kaposi's Sarcoma in Isolated Japanese Islands,” Journal of Infectious Diseases 216, no. 7 (2017): 850–858, 10.1093/infdis/jix424. 28968717

[29] S. C. Dollard , L. M. Butler , A. M. G. Jones , et al., “Substantial Regional Differences in Human Herpesvirus 8 Seroprevalence in Sub‐Saharan Africa: Insights on the Origin of the “Kaposi's Sarcoma Belt,” International Journal of Cancer 127, no. 10 (2010): 2395–2401, 10.1002/ijc.25235. 20143397 PMC2895015

[30] A. Z. Cheng , S. N. Moraes , C. Attarian , et al., “A Conserved Mechanism of APOBEC3 Relocalization by Herpesviral Ribonucleotide Reductase Large Subunits,” Journal of Virology 93, no. 23 (2019): e01539‐19, 10.1128/jvi.01539-19. 31534038 PMC6854502

[31] R. Sanjuán , M. R. Nebot , N. Chirico , L. M. Mansky , and R. Belshaw , “Viral Mutation Rates,” Journal of Virology 84, no. 19 (2010): 9733–9748, 10.1128/jvi.00694-10. 20660197 PMC2937809

[32] K. M. Tamburro , D. Yang , J. Poisson , et al., “Vironome of Kaposi Sarcoma Associated Herpesvirus‐Inflammatory Cytokine Syndrome in an AIDS Patient Reveals Co‐Infection of Human Herpesvirus 8 and Human Herpesvirus 6A,” Virology 433, no. 1 (2012): 220–225, 10.1016/j.virol.2012.08.014. 22925337 PMC3505605

[33] S. A. Roberts , M. S. Lawrence , L. J. Klimczak , et al., “An APOBEC Cytidine Deaminase Mutagenesis Pattern is Widespread in Human Cancers,” Nature Genetics 45, no. 9 (2013): 970–976, 10.1038/ng.2702. 23852170 PMC3789062

[34] S. Li , B. Liu , M. Tan , et al., “Kaposi's Sarcoma Herpesvirus Exploits the DNA Damage Response to Circularize Its Genome,” Nucleic Acids Research 52, no. 4 (2024): 1814–1829, 10.1093/nar/gkad1224. 38180827 PMC10899755

[35] J. C. Santiago , J. D. Goldman , H. Zhao , et al., “Intra‐Host Changes in Kaposi Sarcoma‐Associated Herpesvirus Genomes in Ugandan Adults With Kaposi Sarcoma,” PLoS Pathogens 17, no. 1 (2021): e1008594, 10.1371/journal.ppat.1008594. 33465147 PMC7845968

