# Supplementary material for: KSHV Genome in Saliva, Whole Blood and Kaposi's Sarcoma Biopsy Specimens in Republic of Congo: Phylogenetic Analysis and an APOBEC3B Mutational Signature
Source: J Med Virol. 2026 Apr 30;98:e70933. doi: 10.1002/jmv.70933 (PMC13129949; doi:10.1002/jmv.70933)
Supplement: Supplementary file 1 — Supporting Figure [file JMV-98-e70933-s003.docx]

**Supplementary Figure 1: The coverage and the depth of sequencing for each sample analysed.**


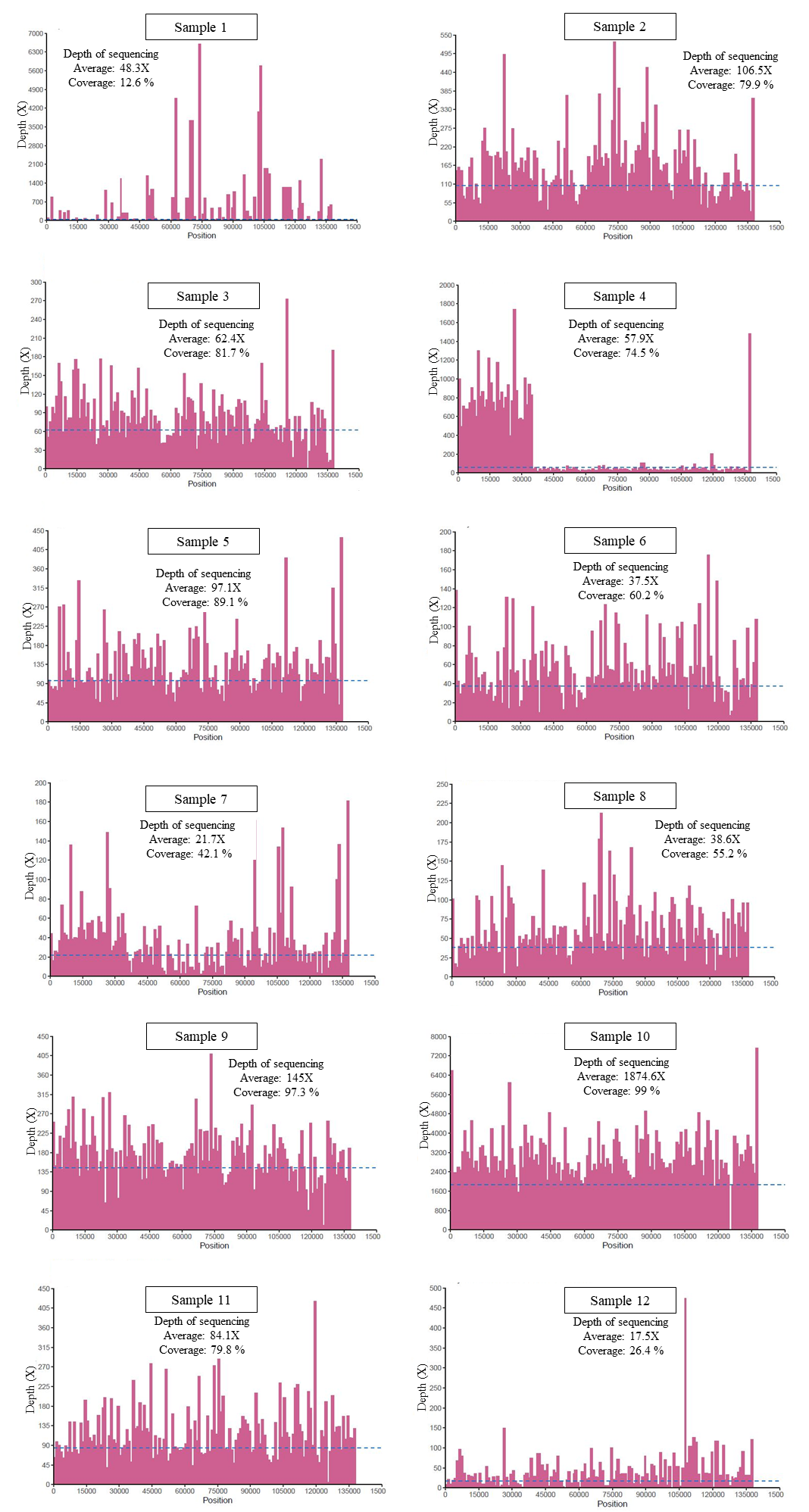


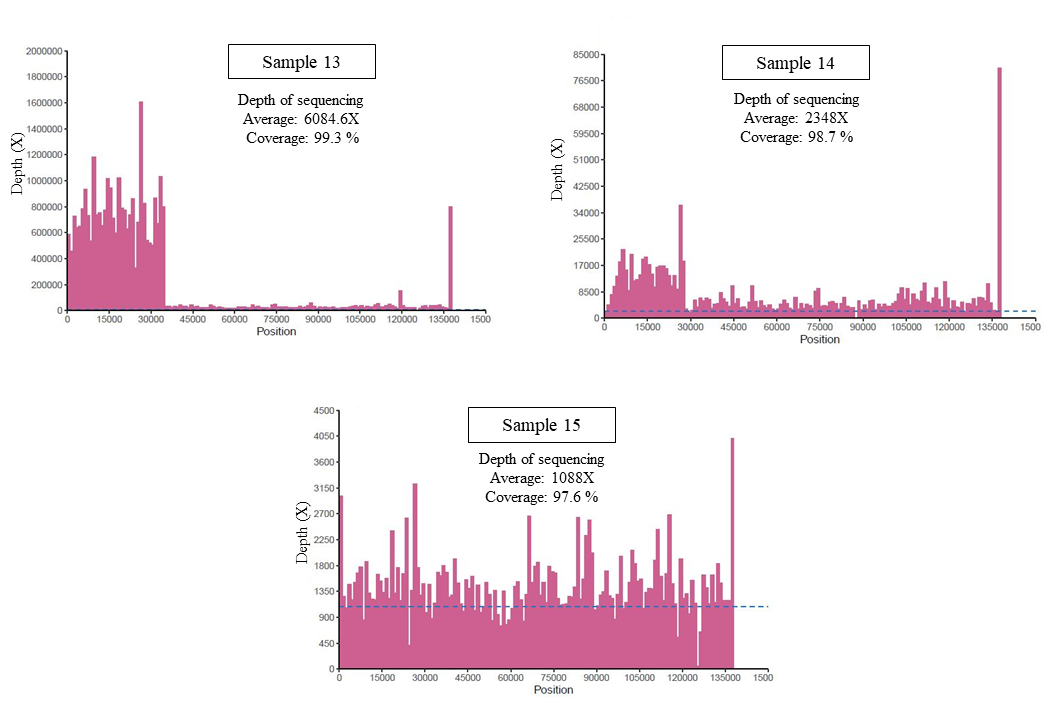

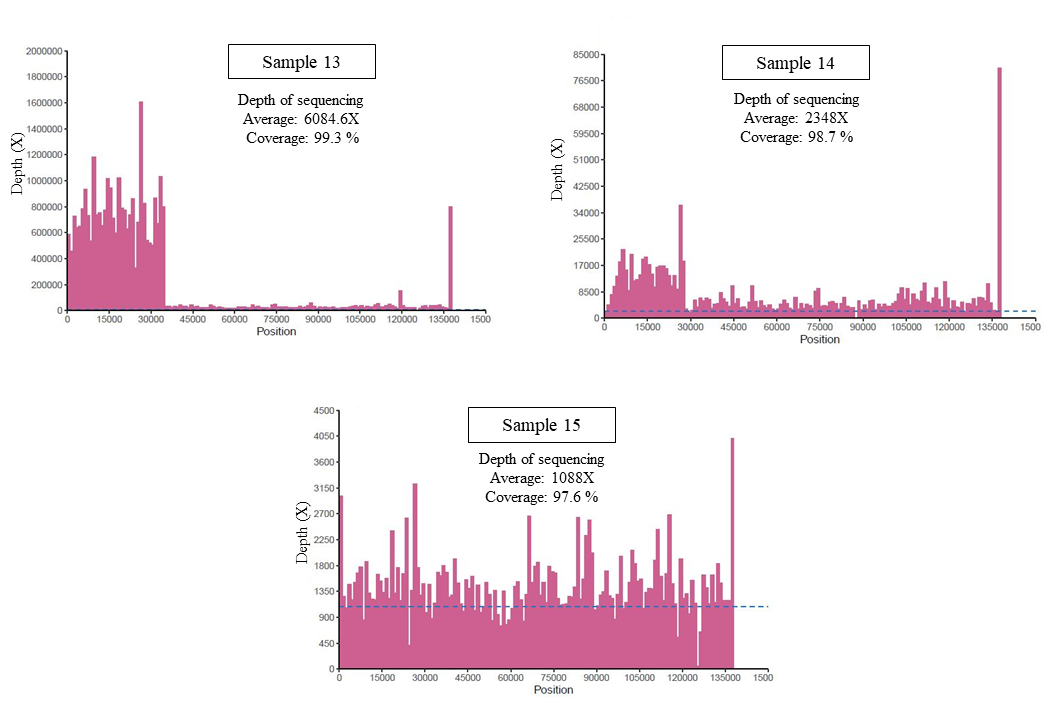


*x-axis: nucleotide position on KSHV genome; y-axis: depth of sequencing: blue dotted line: median of depth sequencing for each sample.*
