# Supplementary material for: KSHV Genome in Saliva, Whole Blood and Kaposi's Sarcoma Biopsy Specimens in Republic of Congo: Phylogenetic Analysis and an APOBEC3B Mutational Signature
Source: J Med Virol. 2026 Apr 30;98:e70933. doi: 10.1002/jmv.70933 (PMC13129949; doi:10.1002/jmv.70933)
Supplement: Supplementary file 2 — Supporting Method [file JMV-98-e70933-s001.docx]

**Supplementary method**

The following part described the different steps performed by our in-house pipeline for consensus sequence reconstruction and variant calling.

Step 1: Base calling of raw fluorescence signals measured for each cluster in the flow cell recorded in BCL format to be converted to FASTQ format. One R1 and one R2 file for each sample (performed directly by the sequencing provider)

- Step 2: Trimming of reads with Trimmomatic using forward and reverse cutting to remove adapter sequences and a sliding window to trim low-quality regions with a cutoff at Q20

- Step 3: Quality control of trimmed reads with Fastqc

- Step 4: Mapping of reads to the KSHV reference (NC_009333) using the Bowtie2 program. The latter works on the principle of Burrows Wheeler Transform (BWT) for mapping. The default parameters were used.

- Step 5: Use of a homemade tool coded in Python to extract information on the quality of the mapping

- Step 6: Calling variants with bcftools mpileup followed by bcftools call, setting ploidy to 1 and filtering variants with a score >=Q20

- Step 7: Searching for regions with low coverage depth < 20X with bedtools genomecov

- Step 8: Building consensus genomes with bcftools consensus using the identified variants and low-coverage regions.
